# Landscape disturbance impacts on *Attalea butyracea* palm distribution in central Panama

**DOI:** 10.1186/s12942-020-00244-y

**Published:** 2020-12-09

**Authors:** Caitlin E. Mertzlufft, Marguerite Madden, Nicole L. Gottdenker, Julie Velásquez Runk, Azael Saldaña, Susan Tanner, José E. Calzada, Xiaobai Yao

**Affiliations:** 1 Geospatial Research, Analysis, and Services Program, U.S. Agency for Toxic Substances and Disease Registry, Atlanta, GA USA; 2 Department of Geography, University of Georgia, Athens, GA USA; 3 School of Veterinary Medicine, University of Georgia, Athens, GA USA; 4 Department of Anthropology, University of Georgia, Athens, GA USA; 5 Gorgas Memorial Institute, Panama City, Panama

**Keywords:** *Attalea butyracea*, Chagas disease, Zoonoses, Remote sensing, GIS, Neglected tropical diseases, Panama

## Abstract

**Background:**

Increased *Attalea butyracea* palm propagation, notable for its role as key habitat for the primary Chagas disease vector in Panama, has been linked to landscape disturbance in single-palm observations in this region. Close proximity of these palms to human dwellings is proposed to increase risk of Chagas disease transmission from sylvatic transmission cycles to domestic transmission involving human populations. This study examines the relationship between landscape disturbance and mature *A. butyracea* spatial distribution, density, and proximity to human populations and vector and reservoir species’ movement corridors at a regional scale in a 300 km^2^ heterogeneous tropical landscape in central Panama.

**Methods:**

We remotely identified the locations of over 50,000 mature *A. butyracea* palms using high-resolution WorldView2 satellite imagery. A local Getis-Ord Gi* spatial analysis identified significant clusters of aggregated palms. Associations between palm and cluster abundance and a landscape disturbance gradient, derived from official Panama land cover data, were tested using Chi-square tests for Homogeneity and Z-test for proportions. Kruskall-Wallis non-parametric analysis of variance tests were run to assess whether palm cluster area varied by disturbance level, or whether disturbance was associated with proximity of palms and palm clusters to susceptible populations or vector movement corridors.

**Results:**

Our findings indicate a regional relationship between landscape disturbance and *A. butyracea* occurrence. We observe a significant increase in both individual and clustered *A. butyracea* in secondary forest, but a reduction of palms in agricultural settings. We do not detect evidence of any reduction in abundance of palms in residential settings. The majority of residential and commercial buildings in our study area are within vector flight distance of potential vector habitat in palm crowns.

**Conclusions:**

We observe probable anthropogenic elimination of *A. butyracea* palms in agricultural, but not residential, settings. Even in heavily deforested regions, significant concentrations of mature palms remain in close proximity to human establishments.

## Background

**Fig. 1:**
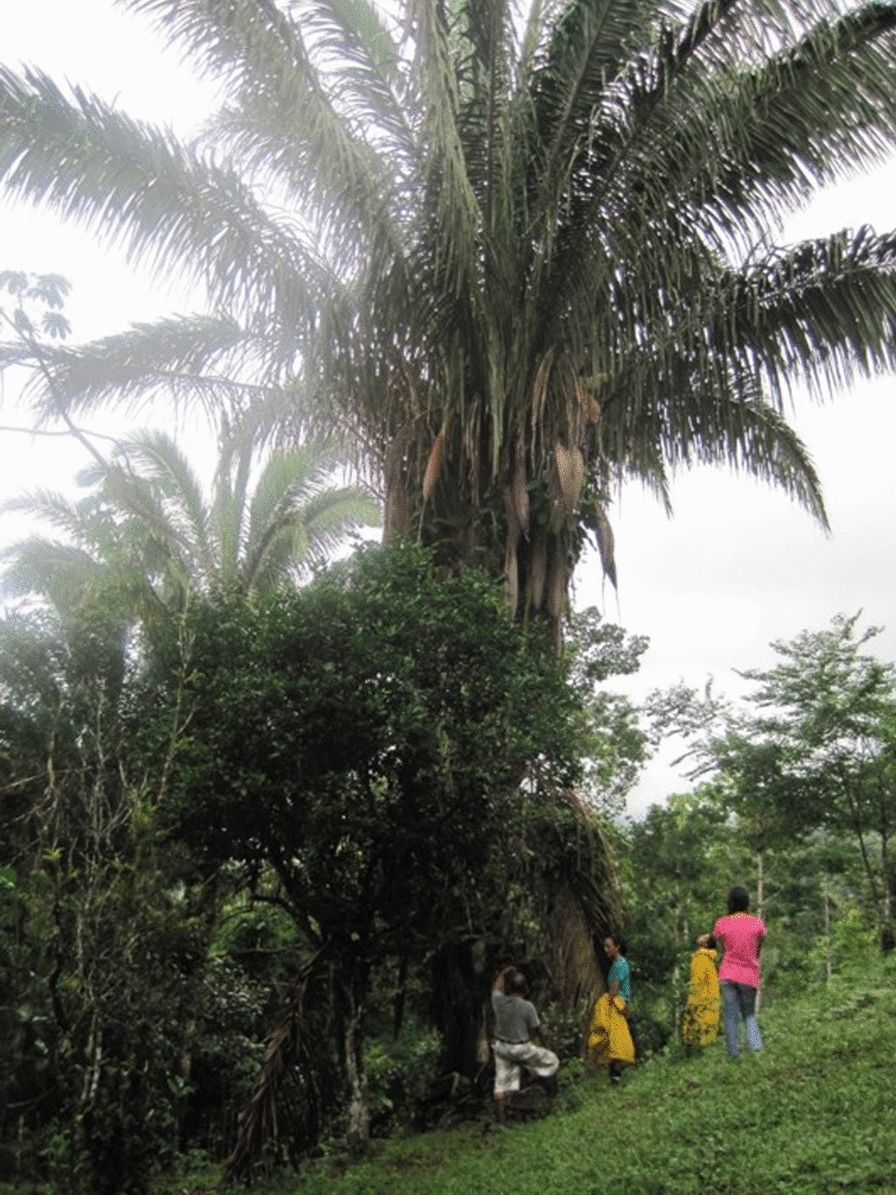
*A. butyracea*, characterized by their large, dense canopies, are the preferred habitat of key Panamanian Chagas disease vector *R. pallescens*

Chagas disease, or American trypanosomiasis, is a vector-borne zoonotic infectious disease endemic to Latin America that is caused by infection with kinetoplastid protozoan parasite., *Trypanosoma cruzi* [1]. The primary vector of Chagas disease in Panama, the triatomine bug *Rhodnius pallescens* (Hemiptera: Reduviidae) [2, 3], preferentially inhabits the palm tree *Attalea butyracea* [4]. The *A. butyracea* palm (Fig. 1) is a dense-crowned species in the subtribe *Attaleinae* that ranges from Mexico to western Amazonia [5, 6]. This palm is characterized by a large crown (individual fronds may reach up to 10 m in length), which contains dense organic material well suited to supporting the microclimate *R. pallescens* bugs require, as well as habitat for the arboreal mammals on which they preferentially feed [4, 6–11]. *R. pallescens* vectors are true palm specialists [7] and *T. cruzi* parasite transmission in Panama is typically confined to sylvatic transmission cycles within palm crowns [12, 13]; however, adult *R. pallescens* in palms near households occasionally invade homes, either in search of potential food sources due to overcrowding of palm canopies [14, 15], or through attraction to electric lights [16, 17]. Therefore Chagas disease transmission in human populations remains a low but constant risk in Panama due to potential crossover from sylvatic to domestic transmission cycles [7, 12]. Close proximity of *A. butyracea* palms to households is considered an elevated risk factor for Chagas disease transmission for this reason [3, 4, 15, 18–21].

*A. butyracea* palms have a propensity to thrive in disturbed landscapes [5, 22, 23], defined here as altered physical environments that disrupt or change the underlying ecosystem [24]. Within Panama, studies of individual *A. butyracea* palms have shown increased seedling survival rates in disturbed habitat due to decreased predation of palm seeds, and suggest that conspecific stands, or clusters, of palms may develop in disturbed habitats due to heighted survival rates of seedlings close to parent trees [25, 26]. This study expands upon individual palm studies to examine the relationship between landscape disturbance and mature *A. butyracea* spatial distribution, density, and proximity to human populations and vector and reservoir species’ movement corridors at a regional scale. Using high-resolution WorldView2 satellite imagery, we have identified a comprehensive subset of over 50,000 mature *A. butyracea* palms within a 300 km^2^ heterogeneous tropical landscape in central Panama. We explore the spatial distribution of this palm sample across a gradient of landscape disturbance, which ranges from areas with limited natural or anthropogenic alteration to areas highly and regularly impacted by human activities: mature forest, secondary and planted forest, pasture, planted food crops, and the built environment. Controlling for other environmental factors known to influence palm distribution [5, 27, 28], (i.e. elevation, precipitation, soil type, average temperature and temperature seasonality) we ask the following questions:

1. Is there a relationship between landscape disturbance and the distribution or density of individual *A. butyracea* palms at a regional scale?
2. Is there a relationship between landscape disturbance and the distribution, density, or area of monospecific stands of *A. butyracea* palms?
3. Is there a relationship between landscape disturbance and the proximity of palms or palm stands to either human populations or movement corridors for Chagas disease vectors and sylvatic hosts (e.g., riparian zones or other *A. butyracea* palm trees)?

## Methods

### Study area

**Fig. 2:**
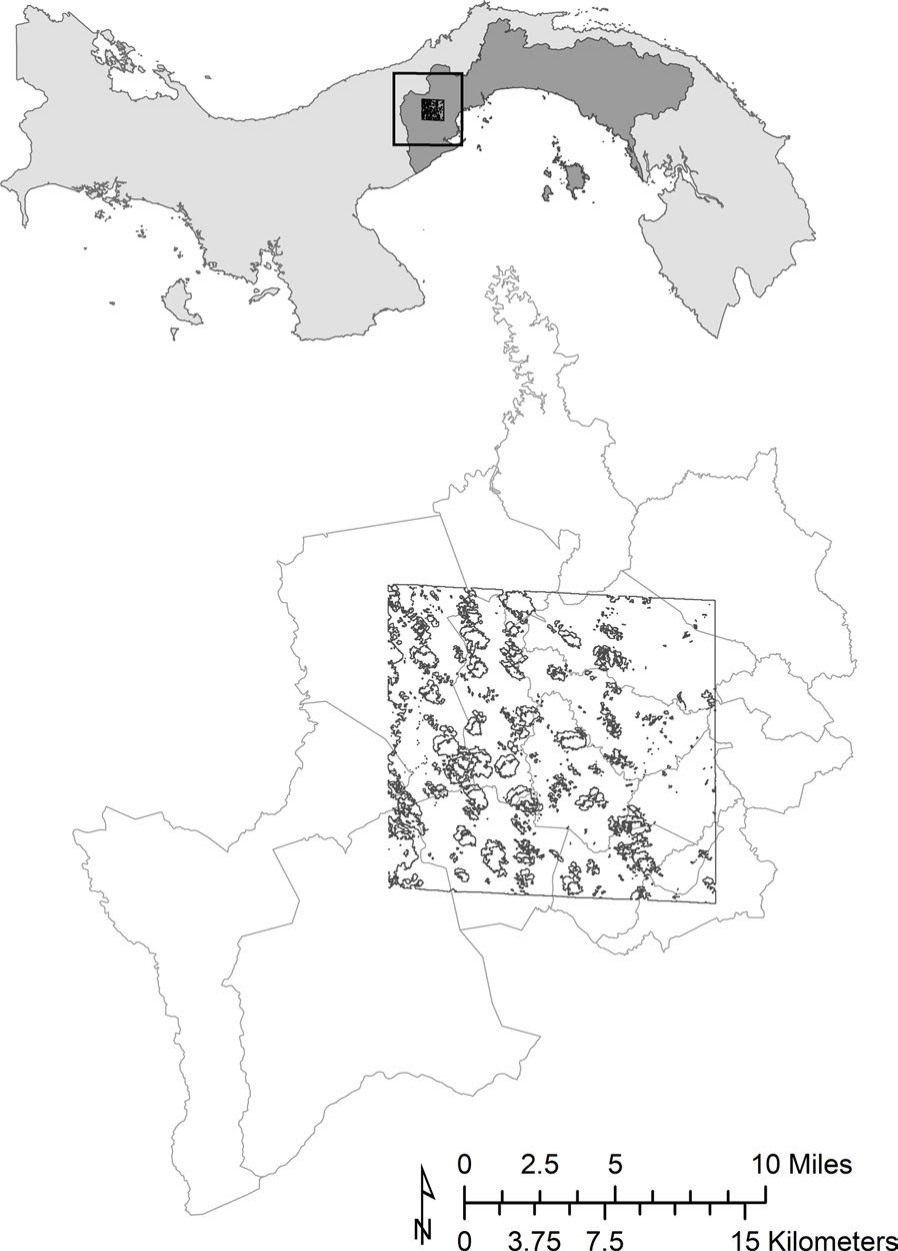
Study area: WorldView2 satellite image footprint, with clouds and shadows masked, includes all or part of 17 corregimientos in the central Panama Oeste district

The study area is located in the Panamá Oeste Province in central Panama, straddling La Chorrera and Capira districts and covering all or part of seventeen *corregimientos* (sub-districts) (Fig. 2). Guided by satellite imagery and the expert regional knowledge of members of our team, we selected this location due to its known history of Chagas disease transmission [12], abundance of *A. butyracea* palms, and adequate representation of each category of our disturbance gradient. Because landscape disturbance is by no means the only variable associated with *A. butyracea* prevalence, we chose an area with minor variation in other environmental variables known to influence palm distribution: elevation, precipitation, soil type and temperature [5, 27–29].

### Data collection and pre-processing

#### Satellite imagery

To conduct our remote identification of *A. butyracea* palm locations, we obtained a high-resolution WorldView2 (WV2) satellite image in both multispectral (blue, green, red and near infrared bands) and panchromatic format covering 300 km^2^ of our region of interest (bounding coordinates NW: − 80.013, 8.94; NE: − 79.854, 8.93; SE: − 79.855, 8.785; SW: − 80.013, 9.793) (DigitalGlobe, Inc., 2017). Our imagery was collected February 1, 2013 at an off-nadir angle of 16.19°, producing a spatial resolution of 2.03 m for multispectral bands and 0.51 m panchromatic. To remove distortions due to image tilt and region topography, the imagery was orthorectified with a NASA Shuttle Radar Topography Mission (SRTM) 30 m digital elevation model of Panama, obtained through the Smithsonian Tropical Research Institute’s (STRI) GIS OpenData Portal.

#### Palm collection and validation

The palm data used in this study were obtained through a combination of manual field sampling in our study area in 2016 and 2017 and remote palm coordinate collection via visual interpretation of the WV2 satellite imagery. Our fieldwork was authorized by the Panama Ministry of the Environment (MiAmbiente), the Gorgas Memorial Institute for Health Studies (ICGES), and STRI.

We recorded the field locations of an initial convenience sample of 131 *A. butyracea* palms in our study region in July 2016. This palm dataset consisted primarily of easily accessible roadside palms, comprising both freestanding individuals and those located within a contiguous forest. We collected coordinate points of each palm with a Garmin Oregon 550 T handheld GPS unit and photos of each palm crown as a record of species identification; coordinates and photos were linked with a unique identifier. We converted the palm coordinates to a point shapefile and overlaid these on our WV2 image, where each point was manually assessed to confirm visibility of a corresponding palm crown within the satellite image. We randomly selected 30% (n = 39) of our palm sample to reserve as a validation subset; the remaining 70% (n = 92) of the sample was used for training to manually digitize *A. butyracea* crowns across the remainder of the WV2 image.

**Fig. 3:**
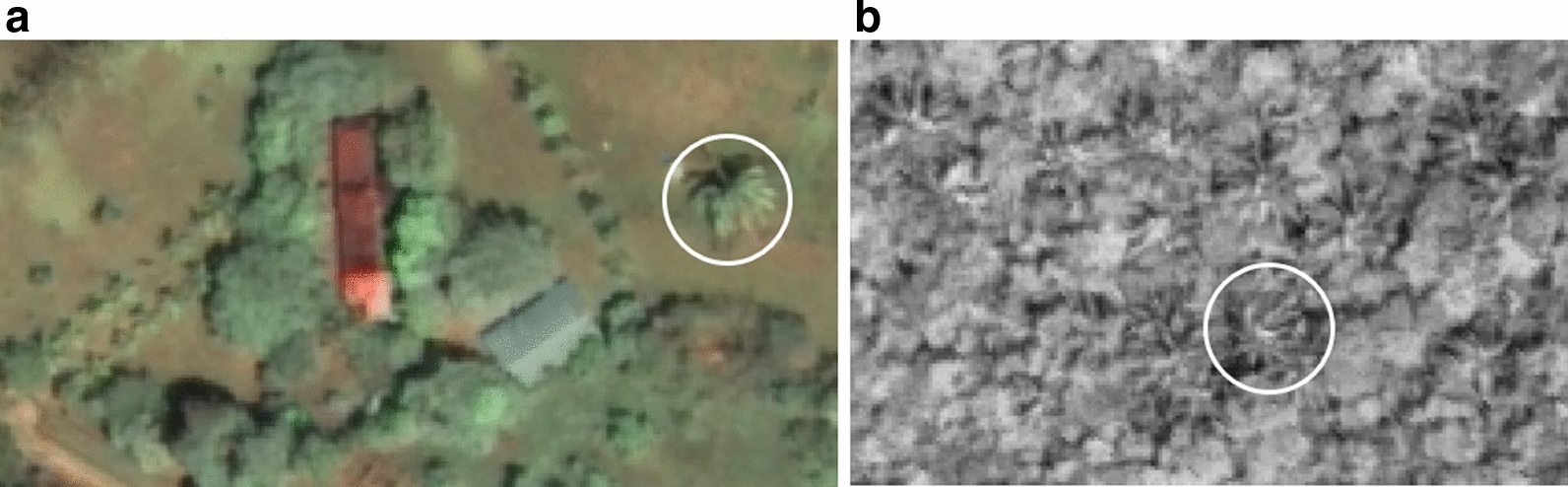
**a** Aerial view of palm crowns in pansharpened true-color WorldView2 satellite imagery. The suspected *A. butyracea* palm (circled) is distinguished from the palms lining the entryway (left) by size, texture, and color. **b** *A. butyracea* palm crowns are clearly visible by their texture and star-shaped crown in dense canopy in the 0.5 m spatial resolution panchromatic WorldView2 imagery. (one of several circled)

For the methodological remote detection of *A. butyracea* palm crowns in our study area, we created a grid across the imagery’s extent, consisting of 535 (0.75 km-by-0.75 km) cells, which was generated by hollow square tessellation in ArcMap 10.5.1 software (ESRI, Redlands, California). Each one of these 0.56 km^2^ cells was carefully analyzed for palm presence using both panchromatic and pan-sharpened (i.e., a fused 2 m multispectral and 0.5 m panchromatic image having both high spatial resolution and four color bands) WV2 imagery. Mature palm crowns are readily distinguished on remotely sensed imagery, due to their distinctive starburst shape (Fig. 3) and characteristically large size [5, 6].

However, to increase palm visibility, each color band of the pan-sharpened image was enhanced with high contrast settings and brightened. The pansharpened imagery was set to a true color display for visualization of palm crowns, which was useful for palm species differentiation, along with scale and texture, (Fig. 3a). Texture showed more clearly on the panchromatic imagery, which was especially useful in recognizing palm crowns in dense, contiguous canopy (Fig. 3b). Beginning with our training dataset and then moving systematically through the grid cells, a trained analyst identified all large-crowned (7–12 mdiameter) palms either fully or partially visible that matched the *A. butyracea* training set’s shading and crown shape/texture characteristics. Palm locations were delineated as points centered on the palm crown and recorded in a point shapefile. Our final dataset totaled 50,955 possible mature *A. butyracea* palms.

**Fig. 4:**
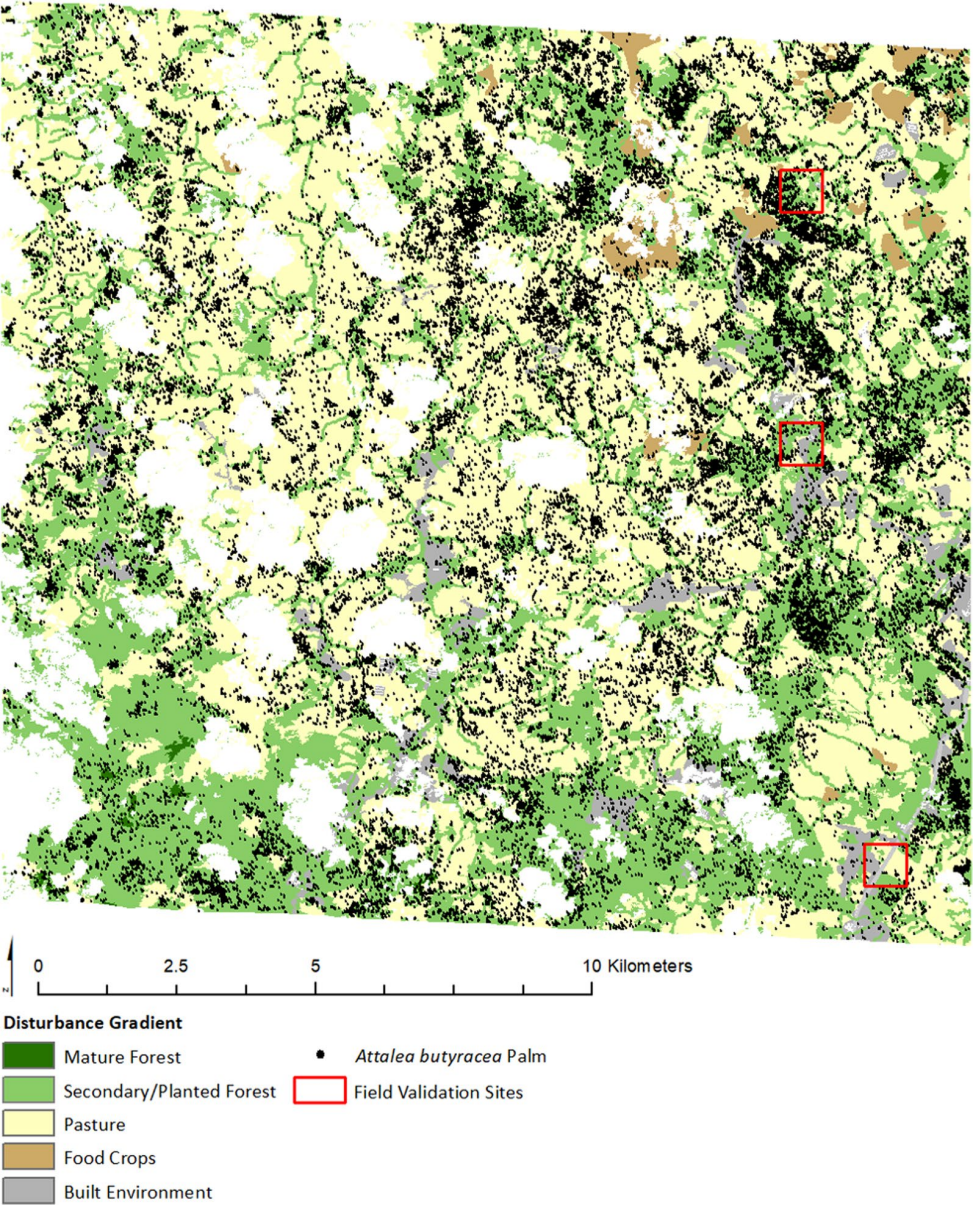
Palm sample and testing sites overlaid on the masked disturbance gradient (clouds and cloud shadow removed). Disturbance categories are derived from 2012 official Panama land cover data

We tested the accuracy of this large dataset both against the reserved validation sample of known *A. butyracea* palm locations and against an additional field sample of 86 *A. butyracea* and non-*A. butyracea* palms collected expressly for this purpose in our study region in late 2017. The locations of this additional fieldwork were chosen at random by selecting three of the grid cells from the shapefile used to organize our systematic remote detection of palms using an online random number generator assigned to the total number of grid cells. However, to ensure both an adequately sized and novel validation sample of remotely sensed palm points, we first identified and excluded grid cells that contained either fewer than 30 remotely identified palm points or any of the 2016 field-derived convenience sample palm locations used initially for our training data. The field validation locations are shown in Fig. 4. At each of these three locations we collected locational coordinates and photos of approximately 30 large-crowned palms, comprising both *A. butyracea* and non-*A. butyracea* palm species, using the same methodology as our 2016 convenience sample. Though our validation field sites were randomly selected, the palms we sampled within them were not. Logistical (e.g., natural and manmade terrain barriers, inability to locate some landowners to request permission to access property) and time constraints made either a truly random sample or a full census of palms in these regions infeasible. Instead, led by the remotely identified palm dataset, we prioritized data collection in accessible areas that contained a combination of pasture and contiguous canopy and were removed from major roadways.

From our 2017 field validation sample, we calculated positive and negative predictive values, as well as sensitivity (true positive rate) and specificity (true negative rate), in order to quantify our ability to remotely detect and properly identify palm species, and to assess likelihood of Type 1 (false positive) and Type II (false negative) errors. We further compared the accuracy of our *A. butyracea* identification stratified by contiguous canopy forest and open pasture. Accurately identified *A. butyracea* (true positives) were palms identified remotely via WV2 imagery that were field verified as *A. butyracea*. Accurately identified non-*A. butyracea* (true negatives) were field-verified non-*A. butyracea* palms that were ignored in our remote sensing effort as “other” species.

#### Disturbance gradient

We derived our natural and anthropogenic landscape disturbance gradient using spatial data from two official Panamanian government datasets: 2012 land cover data and 2010 census data. Both datasets were obtained directly from the ministries responsible for their creation. The 2012 Panama land cover data were generated by Panama’s Ministry of the Environment (MiAmbiente; formerly ANAM) in collaboration with the Food and Agricultural Organization of the United Nations (FAO) [30]. These data were compiled using 5 m spatial-resolution satellite images collected by the RapidEye sensor between January 1, 2011 and April 30, 2012 to form a seamless, 5 m resolution land cover dataset for the entire extent of Panama [30, 31]. Panamanian census data are collected on a decadal basis by the National Institute of Statistics and Census (INEC). The census spatial data used in this study include administrative boundaries of La Chorrera and Capira districts and sub-districts, as well as the locations of buildings, rivers and streams within this region.

The 2012 land cover dataset contains 24 land cover classifications, 11 of which comprise our study region: mixed mature broadleaf forest, mixed mature secondary broadleaf forest, conifer planted forest, hardwood planted forest, new growth/bushes, corn, pineapple, pasture, surface water, populated areas, and infrastructure. We extracted land cover data within the boundaries of our satellite image extent and consolidated similar land cover types to form a 5-category disturbance gradient (Table 1). From least- to most-altered, this disturbance gradient consists of mature forest, secondary and planted forest, pasture, planted food crops, and the built environment. The built environment is a term that encompasses anthropogenic structures and their supporting infrastructure (e.g. roads), to support human activity spaces for living, working, and recreation [32].

**Table 1.** 2012 Panama land cover categories

Original assigned category^a^		Consolidated	Total area		Masked area^b^		Palms	
2012			km^2^	%	km^2^	%	#	%
Bosque latifoliado mixto maduro	Mixed mature broadleaf forest	Mature forest	0.67	0.22	0.53	0.23	125	0.25
Bosque latifoliado mixto secundario	Mixed secondary broadleaf forest	Secondary/planted forest (established)	78.72	26.18	59.50	25.30	16,521	32.42
Bosque plantado de coníferas	Conifer planted forest	Secondary/planted forest (established)	0.03	0.01	0.02	0.01	3	0.01
Bosque plantado de latifoliadas	Hardwood planted forest	Secondary/planted forest (established)	1.04	0.35	0.70	0.30	94	0.18
Rastrojo y vegetación arbustiva	Vegetation regrowth and Bushes	Secondary/planted forest (new growth)	6.52	2.17	5.51	2.34	1593	3.13
Maíz	Corn	Food crops	0.12	0.04	0.12	0.05	1	0.00
Piña	Pineapple	Food crops	6.31	2.10	3.79	1.61	298	0.58
Pasto	Pasture	Pasture	164.02	54.56	130.42	55.45	24,169	47.43
Superficie del agua	Surface water	Secondary/planted forest (riparian)	33.45	11.13	26.43	11.24	7126	13.98
Área poblada	Populated area	Built environment	9.39	3.12	7.86	3.34	1016	1.99
Infraestructura	Infrastructure	Built environment	0.37	0.12	0.34	0.14	9	0.02
		Total	300.65		235.22		50,955	

^a^Land cover categories assigned by the Panamanian Ministry of the Environment (MiAmbiente), formerly La Autoridad Nacional de Ambiente de Panama (ANAM)

^b^There was no significant difference between masked area and total area in any category

A comparison of the 2012 land cover data and our 2013 satellite imagery indicated high consistency between the two datasets. However, we observed poor representation in the land cover data of considerable contiguous tree cover associated with the riparian zones of minor streams and rivers, which may serve as important wildlife and vector movement corridors. To spatially define these riparian zones for inclusion in our disturbance database, we applied buffers to the river and stream locations contained in the 2010 census data, which were more complete than the surface water estimates within the 2012 land cover dataset. We applied a 25 m buffer to all rivers and streams within our study area, based on an average of riparian area widths measured in the satellite image. The land cover within this 50 m wide area was reclassified “riparian zone” and included in the secondary forest category of our disturbance gradient.

Due to the heterogeneity of land cover classes that comprise the secondary and planted forest classification, for some analyses we further stratified this category into riparian zone, recent growth (≤ 5 years), and established forest (> 5 years). Recent growth corresponds to the official Panama land cover classification of *rastrojo*, which refers to the initial stages of secondary forest at 5 years of age or less [31]. Established forest is a combination of mixed secondary broadleaf forest and both coniferous and deciduous planted forests.

Due to cloud cover and cloud shadow, which covered approximately 6% of our satellite image surface, certain regions of the imagery were obscured to remote palm extraction. To accurately calculate the density of our palm sample stratified by our disturbance gradient, it was necessary to mask the land cover data to replicate the visible satellite surface. We first generated a shapefile of the obscuring cloud cover/cloud-shadow using object-based image analysis (OBIA) software, eCognition Developer 8.1 (Trimble Inc., Sunnydale, CA). OBIA is an image classification method that transforms high-resolution pixels into meaningful objects, based on user-defined combinations of size, shape, and band metrics; pixels are first grouped, then joined, into desired categories through user manipulation [33]. This shapefile was used to remove obscured regions in our study area for both the original 2012 land cover data and consolidated disturbance dataset, resulting in two masked subsets that exactly matched the visible satellite imagery extent (see Fig. 4). Using z-tests for proportion comparisons, we assessed whether the masked datasets were representative samples of the total study region for each dataset. For each classification scheme, we found no significant difference in land cover distribution between the original dataset and its corresponding masked subset. We conclude that the masked region used for palm extraction and density analyses is representative of the entire study area; no significant bias was introduced to the analysis by disregarding the approximately 6% of the satellite image obscured by clouds or shadow.

### Spatial analysis of palm data

Natural and anthropogenic landscape disturbance is linked with increased *A. butyracea* propagation at local scales [5]. To assess the relationship between landscape disturbance and palm propagation at a regional scale, we compared palm distribution, density, and proximity to key features across the disturbance gradient within our 300 km^2^ study area. Because landscape disturbance is also associated with increased likelihood of *A. butyracea* forming monospecific stands [10, 25, 26], we assessed the spatial relationship of both individual palms and statistically significant clusters of mature palms, a proxy for monospecific stands, for all spatial analyses. All geospatial analyses were conducted using ArcGIS 10.5.1 software (ESRI, Redlands, California) and all statistical analyses were conducted using SAS 9.4 software (SAS Institute Inc., Cary, NC).

#### Palm clustering

We assessed our *A. butyracea* palm dataset for spatially clustered groupings of points that may indicate a monospecific stand, or cluster, of this palm species. Our palm data consisted only of x,y coordinate information, and the very large sample size and narrow distance increment of interest for clusters (~ 30 m) made traditional point pattern analysis of clustering (e.g. Ripley’s K analysis) prohibitively computationally intensive. Instead, we aggregated palm points to the cells of a 50 × 50 m grid vector shapefile overlaid on our study region, which provides a uniform and comparable measure of palm density across our sample area. We tested grid overlays at spatial resolutions of 5 m (an exact replicate of the underlying landscape raster), 30, 50, and 100 m; however, the 50 m spatial resolution best characterized palm distribution without including too much “empty” space (100 m & 250 m), or creating too many “islands” of non-contiguous occupied cells [30 m]. Additionally, *A. butyracea* propagation literature suggests an average of < 10 m seed migration (by predators who feed on the surrounding fleshy mesocarp) from the parent tree, with occasional migration up to 30 m [34, 35]. We anticipate a cell size of 50 m spatial resolution is large enough to pick up clusters of related palms within a single cell, or among neighboring cells. We filtered unoccupied grid cells from the dataset to limit our assessment of palm clustering solely within the observed palm distribution area.

We assessed overall presence or absence of palm clustering in our study area using a global Moran’s I spatial analysis. To reduce the bias of edge effects in our Moran’s I analysis, we row-standardized the spatial weighting scheme, which proportionally controls the weighting of cells with unequal numbers of neighbors [36]. Specific clusters of occupied cells were identified using the local Getis-Ord Gi* analysis, or commonly called hotspot analysis, with an inverse-distance weighting scheme. Local Getis-Ord Gi* is a popular type of Local Indicator of Spatial Autocorrelation (LISA). Given our large sample size, we applied a false discovery rate (FDR) correction to the hotspot analysis, which applies a more conservative threshold to cluster significance in order to reduce Type-1 errors associated with multiple testing and spatial dependency [37].

#### Palm distribution

Palms and significant palm clusters were assigned a disturbance gradient category based on their location and surrounding land cover. To adjust for any minor spatial disagreement between our land cover data and satellite imagery due to limitations of positional error of the two data sets, which might introduce error when overlaying the palm coordinate data with level of disturbance, we assigned palms a corresponding gradient based on the disturbance category that comprised a majority of area within a 10 m buffer zone surrounding each palm point. Palm clusters were assigned the disturbance category that comprised the majority of their area. We used a Pearson’s chi-squared test of homogeneity to analyze whether our observed palm point and cluster distributions statistically deviated from an expected distribution. Because *A. butyracea* palms are ubiquitous across central Panama and our study area controls for known drivers of *A. butyracea* palm distribution (i.e. soil, temperature, precipitation), we expect our palm and cluster distribution to be generally evenly distributed across the disturbance gradient categories if no relationship exists between landscape disturbance and palm presence at a regional scale. We employed pairwise *Z*-tests for proportions to identify the disturbance category or categories driving statistically significant variances from this expected distribution, where applicable.

We also assessed a random, stratified sample of 150 palm trees (thirty palms from each of our five disturbance gradient categories) to test whether average crown diameter, a proxy of palm age, differed significantly across the disturbance gradient. We used an analysis of variance (ANOVA) test to assess whether average palm crown diameter differed significantly by disturbance type.

#### Palm density

Using the disturbance gradient assigned to each palm and palm cluster, we assessed average density of palms and clusters stratified by disturbance gradient. Palm density was measured as number of palms per square kilometer disturbance gradient. Cluster density was measured as average cluster hectare per square kilometer disturbance gradient.

#### Palm proximity to key features

We assessed the distance of each palm and palm cluster to the nearest feature of interest in each of three categories: buildings, rivers, and other palms or clusters. Palm distance was measured from the center point of each crown. Cluster proximity was calculated between the feature of interest and the cluster’s boundary. Proximity to buildings is used as a proxy of Chagas disease risk to human populations, given concerns of crossover between sylvatic and domestic transmission cycles. Distance to riparian areas and other palms/clusters were considered as potential pathways of either vector or reservoir species’ movement [38, 39].

To statistically compare proximity of palms and clusters to our key features of interest, we employed Kruskall-Wallis analysis of variance by ranks tests [40]. Palms and clusters tended to be quite close to features of interest, with a minority of longer-distanced outliers. This skewed distribution violates assumptions associated with the more common statistical test to compare averages, the Analysis of Variance (ANOVA); the Kruskal–Wallis test is the non-parametric equivalent. Where necessary, we followed this test with a pairwise Dunn’s test for non-parametric post-hoc analysis, using a SAS macro developed by Elliott and Hynan [41].

## Results

### Accuracy of remote *A. butyracea* identification

Of the 30% (n = 39) known *A. butyracea* locations withheld from our 2016 convenience sample for validation testing, we accurately identified 95% (n = 37) as *A. butyracea* palms. The two overlooked palms were both partially obscured by non-*Attalea* contiguous canopy. In one instance, only a partial shadow indicated the palm’s presence. However, we found that canopy cover was not a general barrier to *A. butyracea* identification within this subset; we correctly located and digitized all six additional palms partially or mostly obscured by canopy. The validation subset was added to the total palm dataset used for this analysis, and their identifying characteristics studied for further palm extraction.

In our 2017 field survey used to validate a random subset of our final remotely collected palm sample, we collected coordinate and photographic records for 86 mature, large-crowned palms. Of this validation sample, 64% (n = 55) were *A. butyracea*, while the remainder were non-*A. butyracea* controls. The *A. butyracea* sample was evenly divided between pasture palms (n = 28, defined as solitary individuals with minimal undergrowth and no surrounding contiguous canopy) and canopy palms (n = 27, defined as individuals in the understory or part of contiguous canopy). The majority of the non-*A. butyracea* control palms (n = 26, 84%) were solitary pasture palms. Coconut palms (*Cocus nucifera*) comprised most of the control subset; the remainder comprised a variety of large-crowned pinnate palm species.

**Fig. 5:**
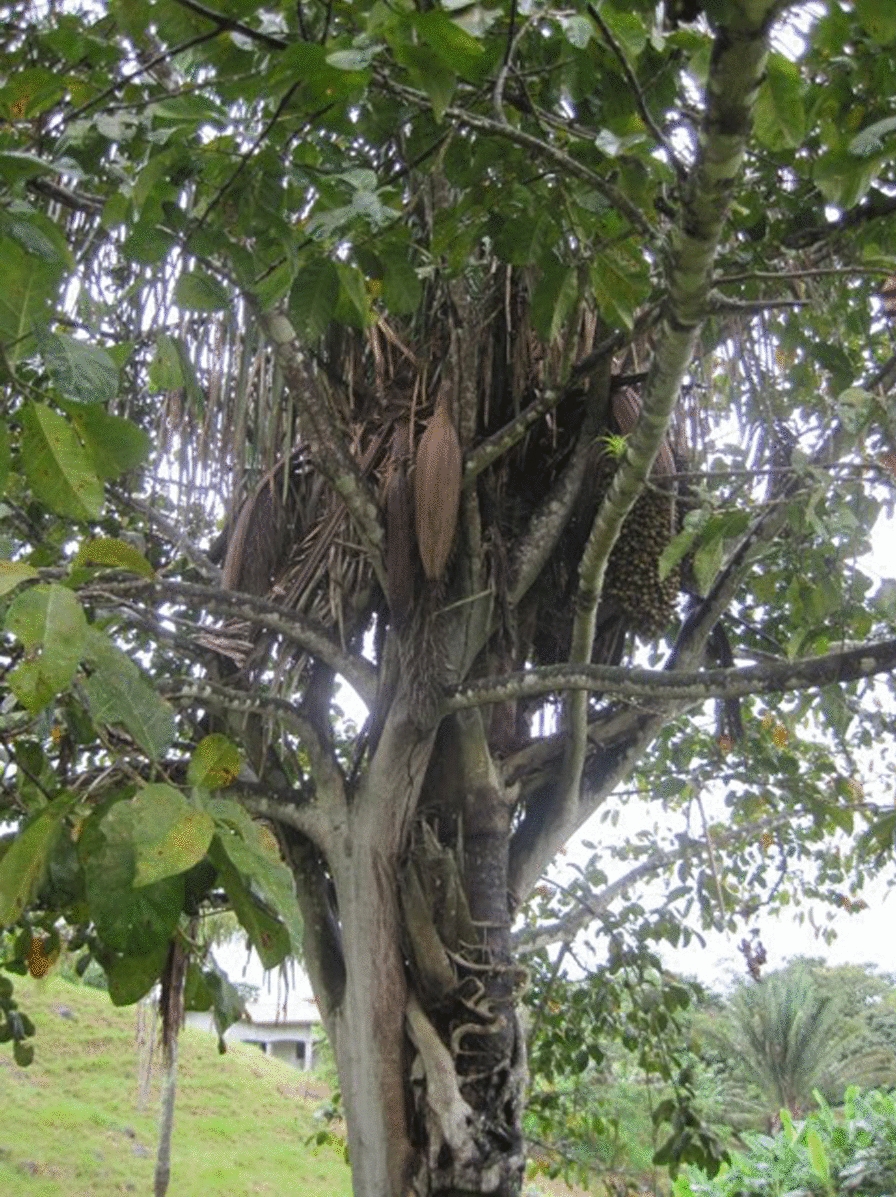
*A. butyracea* palm wrapped in another tree species. Although a solitary pasture palm, this individual’s crown was obscured from satellite imagery by the crown of its encasing tree

Overall, this field sample shows high frequency of correct identification of *A. butyracea* remotely via satellite imagery, with an overall positive predictive value of 80% (Table 2). However, we observed an underestimation of true *A. butyracea* palm presence in our study area, particularly in areas of contiguous canopy: we identified via remote sensing nearly all of the pasture *A. butyracea* palms collected in our field sample (89%), but missed 45% of those collected in canopy areas. Palms obscured from overhead by tree cover were almost always overlooked via satellite imagery. While the majority of the obscured palms were located in contiguous canopy, we observed multiple instances of otherwise solitary pasture *A. butyracea* that were nearly completely encased in other tree species, altering their characteristic crown shape (Fig. 5). We also noted several instances of closely clustered *A. butyracea* palms, particularly along forest edge boundaries, incorrectly marked as a single palm via remote sensing.

**Table 2:** Field assessment of remotely collected palm data

Total field sample *(n* = *86)*				
		Remotely sensed *A. butyracea*		
		YES	NO	Total
Field-verified *A. butyracea*	YES	40	15	55
	NO	10	21	31
	Total	50	36	86
Sensitivity: 0.73 Specificity: 0.68 ^a^PPV: 0.8 ^b^NPV: 0.58				

Pasture sample *(n* = *54)*				
		Remotely sensed *A. butyracea*		
		YES	NO	Total
Field-verified *A. butyracea*	YES	25	3	28
	NO	9	17	26
	Total	34	20	54
Sensitivity: 0.89 Specificity: 0.65 ^a^PPV: 0.74 ^b^NPV: 0.85				

Canopy sample *(n* = *32)*				
		Remotely sensed *A. butyracea*		
		YES	NO	Total
Field-verified *A. butyracea*	YES	15	12	27
	NO	1	4	5
	Total	16	16	32
Sensitivity: 0.56 Specificity: 0.8 ^a^PPV: 0.94 ^b^NPV: 0.25				

^a^Positive predictive value

^b^Negative predictive value

### Landscape disturbance

Our landscape disturbance gradient shows that 99. 8% of our study area has experienced significant natural or anthropogenic alteration; only 0.23% of this region is composed of the least disturbed mature forest (Table 3). However, over a third of the study region comprises secondary forest, which is second lowest on the disturbance index. Nearly six percent of the secondary forest and just over two percent of the entire study area is recent forest growth, aged 5 years or less as of 2012. The majority of the study area (55.5%) is pasture, which is typically reserved for cattle. Food crops and infrastructure combined comprise just over five percent of the region.

**Table 3:** Palm and palm cluster distribution and density by land disturbance gradient

	Land cover		Palms			Palm Clusters		
			Distribution		Density	Distribution		Density
	km^2^	%	N	%	N/km^2^	N	%	N/km^2^
Mature forest	0.53	0.23	125	0.25	235.85	1	0.29	1.89
Secondary/planted forest	92.16	39.18	25,337	49.72^‡^	274.92	173	50.58^‡^	1.88
New growth (≤ 5 years)	5.51	2.34	1593	3.13	289.11	9	2.63	1.63
Established (> 5 years)	60.22	25.60	16,618	32.61^†^	275.95	135	39.47^‡^	2.24
Riparian zone	26.43	11.24	7126	13.98	269.62	29	8.48	1.10
Pasture	130.42	55.45	24,169	47.43^†^	185.32	164	47.95	1.26
Food crops	3.91	1.66	299	0.59^†^	76.47	1	0.29	0.26
Built environment	8.20	3.49	1025	2.01	125.00	3	0.88^†^	0.37
Total	235.22		50,955			342		

^†^*p* value < 0.05 based on *z*-test for proportion compared to associated land cover

^‡^*p* value < 0.01 based on *z*-test for proportion compared to associated land cover

Both pastoral and secondary forested gradients comprise large spaces of contiguous coverage in our study area, but the secondary forest also comprises substantial amounts of forest fragments (Fig. 4). Mature forest is primarily confined to small islands of forest surrounded by large contiguous regions of secondary growth, although in two instances it directly abuts pasture. We see the most forest cover in the more elevated southwest region of our study area.

### Relationship between the distribution and density of individual *A. butyracea* palms and landscape disturbance

Given uniform palm distribution across the landscape, we expect the frequency of palm occurrence to match the proportional breakdown of our landscape disturbance gradient. However, we observe significant divergence in expected distribution of palms in secondary forest, pasture and food crop settings (see Table 3). Nearly half of the palms (49.7%) in our study area are found in secondary and planted forest, which is significantly higher (p < 0.01) than the approximately 40% of the region’s area covered by this gradient type. When secondary forest is further stratified by subtype, we observe that established secondary forest (> 5 years) drives the significance of this category, comprising over 60% of individual secondary forest palms. Palm distribution in secondary forest associated with riparian zones and new growth (≤ 5 years) does not deviate from expected.

In contrast, we observe statistically fewer palms than expected in agricultural settings (p < 0.05). Palms in pastoral and food agriculture zones comprise 47.4% and 0.59% of our total sample, respectively, although these regions account for 55.5% and 1.7% of our study area. We observe the highest palm density in the secondary and planted forested regions, particularly within new growth (≤ 5 years) forest, at 289 palms/km^2^. Palm density is lowest in cropland (76.5 palms/km^2^).

Of particular relevance for Chagas disease transmission, the reduction of palm trees appears confined to agricultural, and not residential, settings. We do not observe any statistically significant reduction in *A. butyracea* presence associated with the built environment, resulting in ample potential vector habitat located near human households. At a density of 125 palms/km^2^, we find over 1000 *A. butyracea* palms within the 8 km^2^ area classified as the built environment, over 95% of which is populated residential and commercial areas.

Additionally, our observations do not support a relationship between landscape disturbance and the average age of palms in this region, as measured through the proxy of palm crown size (Table 4). Our random sample of palm crowns ranged from 4.26–15.97 m in diameter, but their stratified averages did not meaningfully deviate across disturbance categories. However, this finding may also reflect a bias towards the remote detection of larger, mature palms, which our field validation indicated were more likely to be detected in satellite imagery.

**Table 4:** Average *A. butyracea* crown diameter by disturbance gradient

	N	Crown Diameter (m)			
		Min	Max	x̅^a^	sd
Overall	150	4.26	15.97	9.03	2.36
Mature forest	30	5.41	15.97	9.49	2.67
Secondary/planted forest	30	5.65	14.28	9.27	2.29
Pasture	30	5.16	15.00	9.40	2.56
Food crops	30	5.5	12.76	8.09	1.6
Built Environment	30	4.26	14.74	8.89	2.25

^a^There is no evidence of statistical difference among average crown size, given by a one-way analysis of variance (ANOVA)

### Relationship between distribution, density, or area of monospecific stands of *A. butyracea* palms and landscape disturbance

The Global Moran’s I analysis revealed highly significant (*p* value < 0.0001) spatial clustering of mature palms within the broader area of observed palm distribution in our study area. The Getis-Ord Gi* analysis with FPR correction identified 342 statistically significant distinct palm clusters, indicative of monospecific stands of mature palms (Table 3). Given the much smaller sample size, cluster density is far lower than individual palm density throughout the region. We observe the highest density of clusters in established secondary and planted forest (2.24 clusters/km^2^), and lowest densities in areas with planted food crops (0.26 clusters/km^2^) and the built environment (0.37 clusters/km^2^).

The distribution pattern of clustered palms generally mirrors that of individual palm trees. As with individual palms, we observe a statistically significant increase in palm clusters in secondary and planted forest (*p* value < 0.01), which is also driven by significance in the sub-category of established growth (Table 3). However, in contrast to individual palms, we find no association with pasture or food agriculture, but do observe significantly fewer clusters than expected in the built environment (*p* value < 0.05).

Most palm clusters observed in our study region are less than a hectare (ha) in area. The average area of the palm stands remotely identified in this region is 0.42 ha, although the largest single contiguous area with a high density of palms reaches 4.5 ha (Table 5). We find no evidence to support that the average size of palm clusters differs by landscape disturbance level.

**Table 5:** Cluster area by land disturbance gradient

	Cluster area (ha)				
	Total	Min	Max	x̅^a^	sd
Mature forest	0.25	0.25	0.25	0.25	0
Secondary/planted forest	70.50	0.25	4.50	0.41	0.5
New growth (≤ 5 years)	7.25	0.25	4.50	0.81	1.32
Established (> 5 years)	55	0.25	3.75	0.41	0.44
Riparian zone	8.25	0.25	0.50	0.28	0.09
Pasture	69.75	0.25	3.00	0.43	0.43
Food crops	0.25	0.25	0.25	0.25	0
Built environment	1.25	0.25	0.75	0.42	0.24
Overall	142.00	0.25	4.50	0.42	0.47

^a^There is no evidence of statistical difference among average cluster size by disturbance gradient, given by Kruskal–Wallis non-parametric analysis of variance by ranks test

### Relationship between landscape disturbance and the proximity of *A. butyracea* palms or palm stands to human populations or vector/host movement corridors

**Fig. 6:**
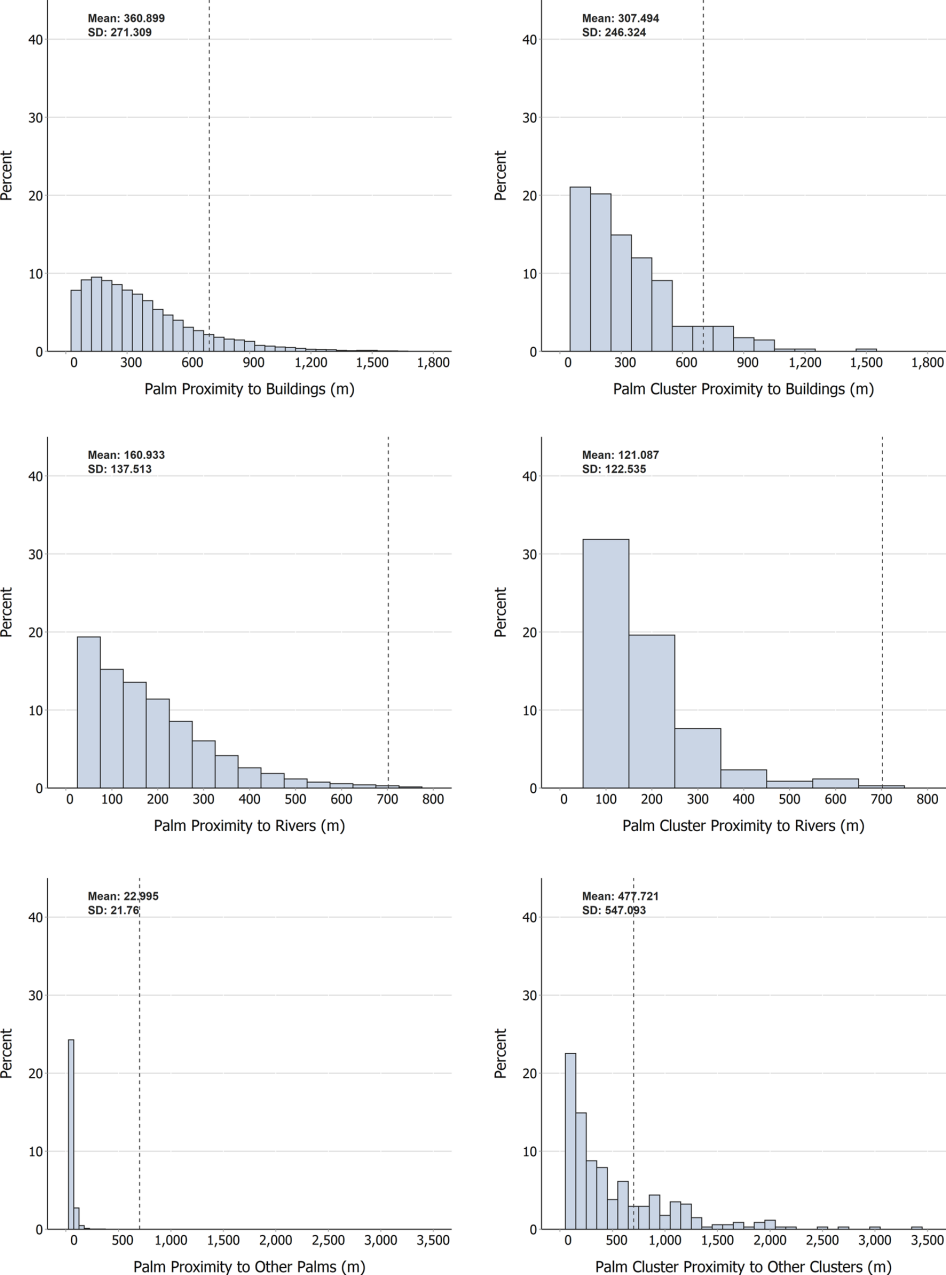
Proximity of palms and palm clusters to features of interest: buildings, rivers, and other palms/clusters. Dashed lines indicate average flight distance of *R. pallescens* vectors (702 m)

Overall, individual palms and palm clusters are heavily skewed toward close proximity to buildings, rivers, and other palms or clusters, but average distance to each of these features varies (Fig. 6). We observed that individual palms are closest to other palms on average, and farthest from buildings. Palm clusters, in contrast, were furthest from other clusters by a large margin, and closest in proximity to rivers. The observed average proximity in all categories is within the recorded average flight distance of *R. pallescens* vectors (702 m [m]) [42].

When stratified by disturbance gradient, Kruskall-Wallis tests indicated no significant change in proximity between clusters of palms in different land covers (*p* value = 0.0799). However, there was a significant association between landscape disturbance and the proximity of palm clusters to both buildings (*p* value = 0.0458) and riparian corridors (*p* value < 0.0001). Proximity of individual palms to other palms, rivers, and buildings were all significantly associated with landscape disturbance per all single palm Kruskall-Wallis tests (*p* value < 0.0001). A post-hoc pairwise analysis using Dunn's Multiple Comparison Test indicated that palm clusters are spaced much farther apart than individual palms, at more than 300 m in all areas but the built environment (average spacing 168.75 m) compared to only slightly over 20 m separation of individual palms on average (Table 6). Individual palms and palm clusters within the built environment are closest to households, at less than 60 m each on average, while those in a mature forest setting are farthest away, at over 8000 m each on average. There is no clear relationship between palm and palm cluster proximity to rivers in association with our disturbance gradient, except those in riparian zones are inherently closest.

**Table 6:** Average distance (m) of palms and clusters to objects of interest

	Buildings		Rivers		Other palm/cluster	
	Palm	Cluster	Palm	Cluster	Palm	Cluster
Mature forest	833.27^a^	1047.6^b^	166.64	0^b^	23.53	403.21
Secondary/planted forest	380.17^a^	322.15	141.79^a^	119.38	22.16	520.75
New growth (≤ 5 years)	358.42	358.87	181.27	180.23	19.83	335.59
Established (> 5 years)	404.53	327.73	193.87	140.94	21.32	550.42
Riparian zone	328.23	284.82	11.51	0.11	24.62	440.10
Pasture	352.34^a^	292.36	182.01^b^	124.03	23.66	438.89
Food crops	263.37^a^	340.62	159.47	130.9^b^	25.96	403.20
Built environment	57.12^a^	31.63^b^	136.77^b^	95.77	27.08	168.75

Comparisons run using Dunn's Multiple Comparison Test for non-parametric post-hoc pairwise analysis, alpha = 0.05

^a^This value is significantly different in all possible pairwise tests

^b^Shared categories are only significantly different from each other in pairwise testing

## Discussion

### Remote sensing of *A. butyracea* palms

Manually censusing the locations of palm trees in the field is a labor-intensive task, particularly over large or remote areas. Where palm location, count, or crown-size are the key variables of interest, many studies have turned to remote data acquisition from satellite or aerial sensors to facilitate palm identification. Our identification of *A. butyracea* palm crowns using high spatial-resolution WV2 satellite imagery is consistent with the challenges and findings of previous tropical studies of remote tree crown extraction for both palm [29, 43] and non-palm tropical tree species [44, 45]. Our field validation survey suggests overall high accuracy (80%) in assigning the correct species to visible *A. butyracea* crowns (low commission error), but frequent underestimation (58%) of true numbers of our target species (high omission error). Inability to observe understory *A. butyracea* crowns obscured by forest canopy or to distinguish closely spaced individual *A. butyracea* contributed to underreporting via remote sensing. Through field validation, we discovered multiple instances of two or more close-growing *A. butyracea* palms in a location where one large *A. butyracea* crown was identified via satellite imagery. It is possible that other very large “single” crowns elsewhere in our study area actually depict closely packed palm clusters.

However, our commission and omission errors are consistent with the manual remote tree crown assessments previously described [29, 43–45]. Notably, despite high omission errors, these previous studies found that remotely detected palm crowns reliably tracked overall spatial patterning of their study species, including clustering, based on full censuses of field-verified palm distribution [29, 43]. Due to the heterogeneous land cover in our study area, we are able to further build upon these studies by comparing remote palm crown identification accuracy in deforested as well as forested settings. Unsurprisingly, given increased visibility of single palm crowns growing in open pastures, we observe increased accuracy in both detecting (decreased omission error) and identifying (decreased commission error) *A. butyracea* crowns in pastoral settings due to less crowding and canopy overlap.

Also consistent with previous studies, we detect a bias in our dataset toward mature palms [46]. We also observed that many of the palms missed in our remote inspection, both canopy and pasture palms, were individuals with relatively sparse crowns—an indication of either young age or heavy harvesting of palm fronds, such as in the use of thatching. Without exception, juvenile palms (palms without mature stalks) were missed altogether, despite our 5 m spatial-resolution imagery. Palm crowns at immature growth stages are generally difficult to visualize even on high spatial-resolution imagery, based on smaller crown size and indistinct morphological characteristics [46]. The bias in our dataset toward large-crowned palms in deforested regions does not necessarily render it unsuitable for locating potential Chagas disease vector populations. There is evidence that *R. pallescens* preferentially inhabit larger, mature *A. butyracea* [47], especially in peridomestic settings [48]. Larger palm crowns contain more organic material to support the microclimate and sylvatic host species *R. pallescens* requires to thrive [4, 7, 8, 13].

### Landscape disturbance and *A. butyracea* palms

Based on land cover, almost all of our study area is considered disturbed, except for the < 0.25% that is mature forest. Within the last century, decreased mature forest in Panama is attributed to expanded cattle ranching and increased development of land by ruralfarmers, although secondary forest cover has increased in recent years [49]. Our findings indicate a regional relationship between landscape disturbance and both individual *A. butyracea* palm and monospecific palm cluster distribution and density. Although we observe statistically significant variation between palms and palm clusters and proximity to both buildings and rivers, these observations likely reflect direct relationships between the location of these features (buildings, rivers) and anthropogenic landscape change. For example, we see a general trend of increasingly closer proximity of palms and palm clusters to human establishments as landscape disturbance increases, which is almost certainly due to a positive correlation between landscape disturbance and human population density. In contrast, although palms and clusters are both skewed towards close proximity to rivers, the absence of a clear pattern of river proximity when stratified by disturbance is likely more indicative of no strong relationship between river location and landscape disturbance.

Among both individual palms and palm clusters, we find that even with a probable underestimation of *A. butyracea* presence in forested regions, based on our field validation findings, we observe a much greater abundance of palms and clusters in established secondary forest than we would expect given the landscape distribution. This finding is consistent with *A. butyracea*’s known propensity to thrive in deforested regions [5, 22, 23] and supports at a regional scale localized studies that link increased likelihood of monospecific stands of *A. butyracea* to increased anthropogenic landscape disturbance and fragmentation [25, 26]. However, our study suggests the relationship between habitat disturbance and *A. butyracea* clustering may be parabolic rather than linear. As expected, we observe minimal clustering of palms in the least disturbed habitat of mature forest (Table 3), which is linked to interspecific competition, reduced available sunlight, and increased seed dispersal patterns associated with higher biodiversity in undisturbed landscapes [25]. However, we also observe significantly fewer palm clusters than expected in our most disturbed habitat, the built environment, perhaps due to regular anthropogenic maintenance. Palm cluster abundance is concentrated in established secondary forest and pasture settings, which are both landscapes that have experienced prior disturbance, but experience little to no day-to-day anthropogenic maintenance.

We also link anthropogenic influence to the significantly fewer individual palms observed in pastoral and agricultural settings. This may be indicative of purposeful elimination of palms associated with agricultural environments. However, given the sheer number and density of palms that remain in pastoral settings, at nearly 200 palms/km^2^, reduction of *A. butyracea* in these regions appears gradual. We observe a gradual reduction of palms in pastoral settings, and a sharp decrease in the number of palms associated with planted cropland. This may be due to competing incentives to produce the best agricultural product: although grasses are inherently the dominate vegetation of pastures, solitary palms and small stands of trees are a common feature in pastoral settings in this region, where they provide shade for cattle and other livestock. In contrast, the two predominant crops in our study area, corn and pineapple, both require full sun to flourish.

Critically, reduction of individual *A. butyracea* does not extend to residential and commercial areas within our study region. Even in heavily built-up environments, we find significant numbers of mature *A. butyracea* in close proximity to commercial and residential structures. We do not have sufficient evidence to determine whether this observation corresponds with purposeful maintenance of these palms for their goods and services, as has been recorded elsewhere in this species’ range [6, 50]. However, *A. butyracea* is a long-lived species that matures slowly [6, 9, 10], and the identification of substantial mature individual palms (n = 1593) and nine clusters of mature *A. butyracea* in new forest growth less than 5 years old suggest that palms are retained or otherwise survive forest clearing in at least some areas (Table 3). Further research is needed to assess why mature *A. butyracea* palms in otherwise deforested areas remain in pastures and in close proximity to households, and to assess whether this occurrence is accidental or purposeful. In our fieldwork, we observed several instances of these palms used for household thatch and other services, but a more comprehensive study to understand the social context of this palm in Panama is warranted [51].

### Implications for Chagas disease transmission

Currently, no vaccine exists for Chagas disease, and treatment, where accessible, can result in negative side effects; prevention of initial infection by deterring vectors’ access to susceptible human hosts is the best-recommended strategy for reducing morbidity and mortality related to this disease [18, 52]. Although presence of *A. butyracea* does not guarantee corresponding presence of Chagas disease vectors or the pathogen, because of the vector’s affinity for this palm species, quantifying *A. butyracea* response to landscape disturbance and this species proximity to households may provide new insight into Chagas disease transmission risks in changing landscapes [4, 18]. Actual risk of Chagas disease transmission almost certainly varies within our study region: variations among housing structure, human interaction with palms, vector infestation of palms, and localized presence of sylvatic host species and *T. cruzi* pathogen will all influence individual risk of contracting Chagas disease. However, as informed solely by close proximity to an *A. butyracea* palm, our analyses suggest that most people residing and visiting commercial establishments within our study area are at increased risk of Chagas disease transmission. *A. butyracea* palms are common throughout the populated regions of this study area, at a density of 125 palms/km^2^ within the built environment (Table 3). We observe that the majority of buildings in our study area are within the average flight distance of *R. pallescens* (702 m) [42], which reflects findings seen elsewhere in central Panama [3]. We also find that it is likely within the ability of these vectors to move between sylvatic and riparian movement corridors in this region as needed. The observed palm-to-palm or palm-to-riparian area routes of travel occur at even shorter distances from palms than buildings, which may provide movement corridors for both vectors and their preferred blood meal species [11, 38, 39]. However, more research is required to determine whether movement corridors are protective (by deflecting vectors to preferred hosts) or increase transmission risk for certain human populations.

Our observations provide evidence that palm abundance is reduced in agricultural settings, but not meaningfully reduced or expanded in residential regions. However, *A. butyracea* palms in otherwise deforested peridomestic environments often harbor larger vector populations than their forested counterparts [53]. Additionally, these peridomestic vector populations may be more prone to fly in search of new hosts as their preferred sylvatic host species decrease due to habitat loss, which has been implicated in increased domestic presence of sylvatic vectors in other Latin American countries [54, 55]. Therefore, increased propagation of *A. butyracea* in disturbed environments may pose a threat of increased Chagas disease transmission risk due to a greater abundance of vector habitat, but this is likely a long-term and low-risk threat given this species’ slow growth to maturity. In contrast, mature palms retained during forest clearing arguably pose a more immediate threat of Chagas disease transmission to human populations.

The spatial and temporal randomness of sylvatic triatomine entry to domiciles renders indoor insecticide spraying an economically inefficient barrier in this region, and control of triatomine bug populations in their native environment is difficult without additional harmful ecological effects [7]. Interwoven social and ecological processes complicate the deceptively straightforward solution of spatially distancing *A. butyracea* palms and human populations. As this and other studies indicate, *A. butyracea* palms thrive in disturbed habitats near human settlements, and mature *A. butyracea* palms are socially valued regionally for a number of goods and services, including household thatch, palm wine, medicine, and shade for livestock [56]. These useful properties lead some people to purposefully maintain *A. butyracea* in pasture or nearby residential or commercial areas, even, occasionally, in otherwise clear-cut environments [50]. All of these factors increase the risk of interaction between susceptible human populations and infectious vectors, which may heighten risk of crossover from sylvatic to domestic Chagas disease transmission cycles.

### Limitations

Although we observe a relationship between *A. butyracea* distribution, density and landscape disturbance, we have limited information on the age of our palm sample or on the disturbance timeline within our study area; we are assessing a “snap-shot” in a dynamic process of landscape alteration and vegetation (re)growth. Although additional official Panamanian land cover datasets exist for years 1992 and 2000, they were derived from 30 m spatial resolution Landsat imagery, and are not directly comparable to the 2012 data due to their coarser resolution [31]. The 1992 and 2000 datasets, which are directly comparable, indicate significant deforestation in the Capira District and slight reforestation in the La Chorrera District during this 8 year period; both regions were described as less than 6% forested in 2000 [31].

A brief comparison of the 1992 land cover to our palm data suggests presence of mature palms in recently (< 20 years) deforested regions, which may further indicate survival or purposeful maintenance of mature palms in otherwise cleared landscapes. Because of their large size and slow growth, mature palms are presumed to be > 20 years of age, therefore predating the 1992 data [26]. However, with the scale discrepancy between the datasets, it is impossible to verify whether this observation is a true occurrence or an artifact of the lower-resolution imagery.

Any suggestion of purposeful maintenance of palms, however, is an indication that a better understanding of the social context of *A. butyracea* palms is critical to fully understand the risk of Chagas disease transmission in this region. Further research is needed to comprehensively assess whether social practices in Panama, as in other areas, influence the retention of this species in otherwise deforested peridomestic environments, and whether these practices vary spatially. The high density of *A. butyracea* palms observed in our study area is not consistent throughout all parts of the country. A better understanding of the interwoven social and ecological factors that influence palm prevalence and distribution will facilitate identification of hotspots of current and potential vector habitat. This will aid the production of more targeted Chagas disease prevention and mitigation strategies in this region.

## Conclusion

Our findings support previous studies linking *A. butyracea* abundance to landscape disturbance, as well as those that advise the use of high-resolution satellite imagery as a method of palm detection. It is clear from the distribution, density, and proximity of these palms to both human settlements and natural movement corridors that the potential of *A. butyracea* as a source of infectious Chagas disease vectors is widespread in rural settings. We observe a positive relationship between landscape disturbance and *A. butyracea* palm prevalence in secondary forest. However, we observe a probable anthropogenic reduction of *A. butyracea* palms in agricultural, but not residential, settings. Even in heavily deforested regions, significant concentrations of mature palms remain in close proximity to human establishments.

## Data Availability

The datasets used and/or analyzed during the current study are available from the corresponding author on reasonable request.
